# Evaluation of the Bone-ligament and tendon insertions based on Raman spectrum and its PCA and CLS analysis

**DOI:** 10.1038/srep38706

**Published:** 2017-01-31

**Authors:** Yang Wu, Yu Dong, Jia Jiang, Haiqing Li, Tongming Zhu, Shiyi Chen

**Affiliations:** 1 Department of Orthopaedic Sports Medicine, Huashan Hospital affiliated to Fudan University, No. 12, Wulumuqi Zhong Road, Shanghai, 200040, China; 2 Department of radiology, Huashan Hospital affiliated to Fudan University, No. 12, Wulumuqi Zhong Road, Shanghai, 200040, China; 3 Department of neurosurgery, Huashan Hospital affiliated to Fudan University, No. 12, Wulumuqi Zhong Road, Shanghai, 200040, China

## Abstract

Injuries to the Anterior Cruciate Ligament (ACL) and Rotator Cuff Tendon (RCT) are common in physically active and elderly individuals. The development of an artificial prosthesis for reconstruction/repair of ACL and RCT injuries is of increasing interest due to the need for viable tissue and reduced surgically-related co-morbidity. An optimal prosthesis design is still elusive, therefore an improved understanding of the bone-soft tissue interface is extremely urgent. In this work, Raman spectral mapping was used to analyze, at the micron level, the chemical composition and corresponding structure of the bone-soft tissue interface. Raman spectroscopic mapping was performed using a Raman spectrometer with a 785 nm laser coupled to a microscope. Line-mapping procedure was performed on the ACL and RCT bone insertion sites. The classical least squares (CLS) fitting model was created from reference spectra derived from pure bone and soft-tissue components, and spectral maps collected at multiple sites from ACL and RCT specimens. The results suggest that different source of interface shows different boundary, even they seems have the same components. Compared to the common histology results, it provided intact molecular information that can easily distinguished some relative component change.

Two of the most debilitating and common sites of injuries are Rotator Cuff Tendon (RCT) of the shoulder and Anterior Cruciate Ligament (ACL) of the knee. Damage or Injury of RCT or ACL can cause significant mobility problems and makes a tremendous difference in the quality of life. The most common tendon/ligament related injuries is RCT tears that who involved in physically active individuals as well as elderly people^1^. Surgical treatment is often required and allows for possibly complete recovery, however, structural/mechanical failure rates at the surgical site/of the graft have been reported vary from 20–94%^2^. Meanwhile, ACL injuries are the most frequently occurs to people at a younger age, particularly those with highly active lifestyles^3^. ACL reconstruction requires surgical intervention of treatment and complete recovery requires for the graft ligamentization^4^. ACL reconstruction surgery typically requires the use of autogenous or allogeneic tissue to replace the torn ACL. However, the rate of healing of the ACL reconstruction site is essentially unsatisfactory, the overall ACL failure rate was reported to be 11.9% (range, 3.2% to 27%)^5^. To better understand the mechanism of the failure of tendon/ligament surgeries and to help compose improved graft materials and repair techniques, we find a way to for investigating the ligament and tendon structure by using Raman spectroscopy.

Raman spectroscopy (RS) is a vibrational spectroscopic method based on the inelastic scattering of photons, in which the photons from a monochromatic excitation light source undergo specific frequency shifts related to chemical bonds^6^,^7^. The RS technique utilized in this work takes advantage of the loss of energy of the sample known as Stokes scattering, and is the most common Raman scattering used for analytical investigation^8^. RS provides information about the chemical and structural composition of a sample, meanwhile it has minimal background of water to get rid of complicated pretreatment of samples and almost no damage to the samples as known a noninvasive analytical method^9^,^10^. The RS especially advantageous for the investigation of biomolecules in living tissues compose of up to 70% water^11^. In recent years, with the advent of advanced optical components, as well as advanced software for rapid data processing, applications of RS for the evaluation of biological samples in a variety of biomedical-related disciplines attract more and more attention.

And the former research used the 532-nm laser to analyze the interface. However, 532-nm excitation can cause sample background fluorescence, which may swamp the Raman signal^11^. Fluorescence occurs most commonly in complex organic molecules as found in polymers, numerous synthetic products, and dyes. Our research had adopt the 785-nm laser, because 785-nm has been found to be optimal for these applications, as it largely avoids fluorescence but still returns a Raman signal sufficient to be detect by a CCD at a reasonable SNR (Signal to Noise Ratio)^12^. Wopenka *et al*. used RS to monitor the distribution of minerals and the degree of mineralization across the tendon-bone insertion site in the shoulders of rats. In their research spectra were only acquired from discrete points across the site of tendon insertion into the humerus^13^. Tendon-to-bone integration is a great challenge for tendon or ligament reconstruction regardless of use of autograft or allograft tendons. The artificial ligaments are more and more popular because of the merit of the designability. Although it not harmful per se, but is dangerous when taken with mismatched gradient.

For better understanding of the insertion point’s composition dynamics change, in our work, line-mapping analysis was used to collect the data from the RCT insertion and also from the ACL femoral insertion.

## Results and Discussion

### The histology results

**Figure 1:**
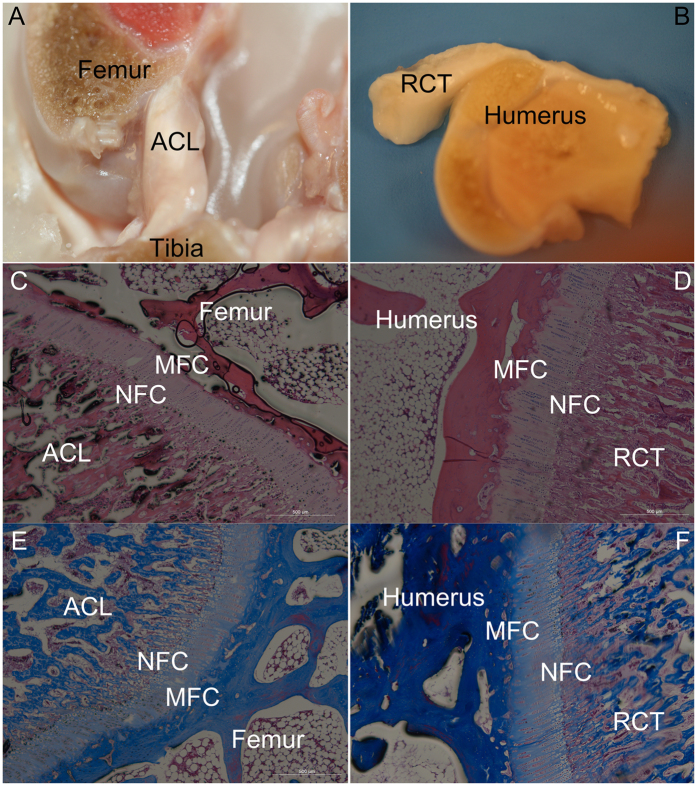
The gross observation & hisotology images. The ACL (**A,C,E**) and RCT (**B,D,F**), (**C,D**) H&E Staining; (**E,F**) Masson Staining; NFC: non-mineralized fibrocartilage; MFC: The mineralized fibrocartilage Magnification 50×.

As shown in Fig. 1(C–F), both of the insertions show four layers: ligament/tendon district contains fibroblasts within a matrix rich in collagens I and III. The non-mineralized fibrocartilage (NFC), directly adjacent to the ligament, is composed of fibro chondrocytes in a matrix of collagens I and II. The mineralized fibrocartilage (MFC) then connects directly to subchondral bone which composed by collagens I and II matrix. The bone that contains osteoblasts is composed a matrix rich in collagen I and hydroxyapatite.

This region-dependent matrix heterogeneity is postulated to permit a gradual increase in stiffness across the interface regions, thereby minimizing stress concentrations and allowing for effective load transfer from ligament to bone.

### The components analysis based on the Raman spectroscopy

**Figure 2:**
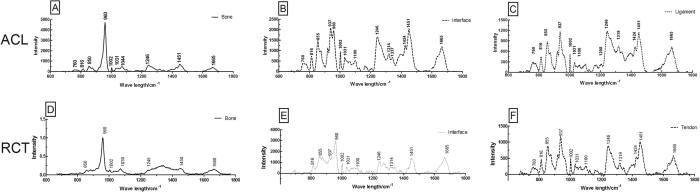
The spectrum of the ACL and RCT insertions. The upper (**A–C**) shows the spectrum of the ACL insertion, the bottom (**D–F**) shows the spectrum of the RCT insertion. Those two different sites present the similar spectrum.

The spectra of the Raman maps (Fig. 2A,B and C) collected from the bone, interface and ligament, matching to the areas shown in Fig. 1. All spectra have common collagen bands, which Raman spectrum is provided for further analysis. A list of the peaks observed in the spectra is shown in Table 1.

The comparison of the spectra revealed that the 1002, 1245, 1450, 1665 cm^−1^ peaks remained exist. The 1002 cm^−1^ represents the phenylalanine (collagen assignment). 1245 cm^−1^ arises from the N-H bending of the peptide group presents the Amide III (of collagen). The CH_2_ deformation presents by a Raman peak at 1450 cm^−1^, bending modes of methyl group (one of vibration mode of the collagen). The amide I of collagen presents at the peak of 1665 cm^−1^ because of the structure of the υ_s_(C=O). The amide III band at 1245 cm^−1^, and the amide I band at 1665 cm^−1^ presents of collagen. For the bone, the *V*_*3*_phosphate vibrations are visible at 1030 and 1044 cm^−1^ 14. However, the *V*_1_ phosphate vibration at 960 cm^−1^ is the strongest marker for bone mineral, whereas the bands at 960 cm^−1^ is typical of hydroxyapatite.

Since collagen and hydroxyapatite are the main constituents of the interface and their bands have already been reported in Raman spectra collected from insertion.

In typical interface, these bands can be clearly seen in the spectra. The intensity of those peaks change with different areas. Diminution of the intensity at 960 cm^−1^ was seen at the transition from the bone to the ligament, the amide bands, which represent collagen, dominated the whole spectra finally (Fig. 2).

Two sharp bands at 937 cm^−1^ and 960 cm^−1^ were both observed in the interface in Fig. 2. The 937 cm^−1^ band represents the vibrations of the collagen backbone and attributes to the presence of the ligament. Variations of Raman intensity within the amide I region suggest the occurrence of the band with the intensities at 1665 cm^−1^, to different secondary protein structures. The two different sources of insertion spectra showed similar Raman spectra.

### The slope of the Raman spectroscopy varies from different insertions

**Figure 3:**
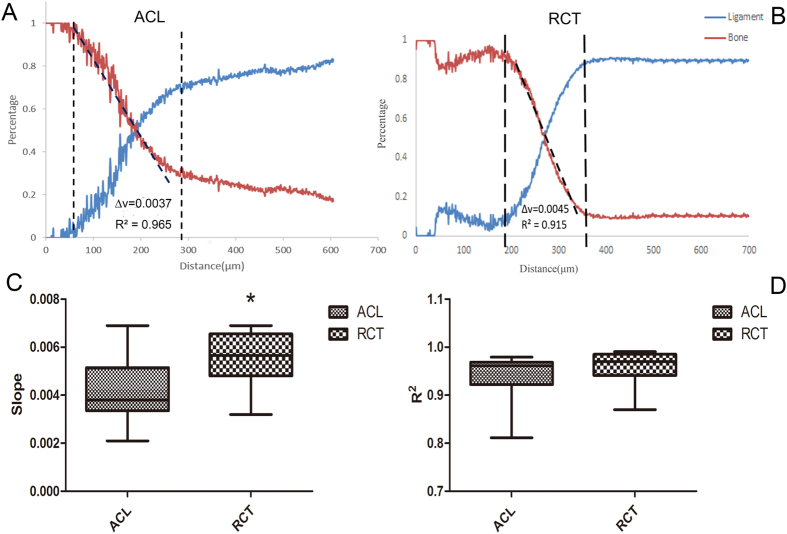
The schematic of how the slope was obtained based on the results of CLS Fitting both by ACLs and RCTs. The results of the comparison of the ACL and RCT slopes and R2, *stands for p < 0.05.

Compared gradient changes of the tissues are shown in Fig. 3A and B. The ACL slope of the mineral changed by 4.139 ± 0.303 × 10^−3^, N = 18; the RCT altered by 5.478 ± 0.295 × 10^−3^, N = 18. The gradient of the mineral was shown to be significantly higher in RCT (p = 0.0032, <0.05) compared to ACL (Fig. 3C). The coefficient of determination (R^2^) for ACL and RCT did not show that the two groups had significant difference (0.9393 ± 0.011; 0.9607 ± 0.0074, N = 18 p = 0.1175, >0.05) (Fig. 3D).

Based on current research, the interface of the tendon/ligament attached to bone varies from the component to the biomechanical. It seems not only just two simple components alteration but also many different molecules evolution. In this study, both the ACL-femur and the RCT-humerus insertions showed the similar spectra as a consequence of the same composition. However, the intensity varied depending on the different areas.

The gradients of two major components were measured by the slope. The result shown that there were significant difference between the two different parts of interface. Although many researchers showed that the insertion of the bone and ligament based on the different methods, the Raman has shown its unique characteristic advantage especially in molecular level. The alterations gradient of the two parts of the tissues may cause by vary of the width of the insertion. The RCT shown more dramatically change compared to the ACL insertion. The information obtained points out that the prosthesis should be customized for different tissue.

The tendon-to-bone insertion combines two different tissues via a gradual transition in composition, mechanical properties, and structure. The surgical repair outcomes are poor till now, besides some surgical techniques, all point to the repair does not mimic the natural attachment.

To date, more and more researchers focus on this area to try to identify the mechanical and physiological functions and compose the biomaterials to mimic the nature structure of insertion^15^. Some researches use osteotendinous scaffolds, which contain opposing gradients of mineral content to mimic the nature insertion^16^.

However, the best design should be based on the fully understanding the target components. Liu *et al*. shown that stress concentrations can be reduced by a biomimetic grading of material properties^17^. Therefore, to better understanding the purpose structure components are more important.

## Conclusion

These data can be used 1) to understand what how the structure of the components change; 2) to compare the components’ change based on these parts.

So, all that can data be used to mimic the biomimetic prosthesis to fulfill the ideal concept that was introduced by L. L. Hench^18^. Those data not only affirmed the former results of the RCT-bone interface, but also provided the major information of the gradient of the insertion. Also, those data not only deliver the ratio of the different components change, but also testified that the diverse interface of vary gradients. All those give the following prosthesis designer powerful evidence to make the real biomaterials for the synthetic ligament.

## Material and Methods

### Preparation of the samples

All experimental procedures were approved by the Shanghai Medical School of Fudan University Animal Care Committee and were conducted in accordance with standard animal research practice. Ligament/tendon-bone samples are removed from the knees and shoulders of three skeletally mature fresh frozen New Zealand rabbits. The intact ACLs and Supraspinatus tendons were isolated. Phosphate-buffered saline was used to keep the tissue samples hydrated during Raman spectral data collection. ACL with intact femoral and tibial aspects were excised from knee joint leaving the entire ligament and bone insertion site intact. The tissue was excised such that the bone was RCT with intact aspect of the humerus were also excised allowing the midline of the interface of the tendon and bone to be prepared as the surface for further evolution. All tissue samples were sawn across the insertion of the ligament/tendon-bone using a diamond wafer blade (Fig. 1A,B).

### Histology study

The complexes (n = 2) were prepared for histological analysis of the ligament-bone interface. Immediately after sacrifice, the graft-bone complex samples were fixed in 10% formalin for 24 h and then embedded in undecalcified in methyl-methacrylate compound, according to established protocols. The complexes were sectioned into layers with a thickness of 5 μm using a Microtome (SM2500, Leica, Germany). The sections were subjected to Hematoxylin and Eosin (H & E) stain and Masson stain. The slides were inspected with a light microscope (Olympus Co., Japan). Digital images were taken using a DP Manager (Olympus Optical Co., Japan).

### Raman spectroscopy and mapping

Raman spectra for development of line, 2-D maps were obtained by using a Horiba Jobin Yvon Xplora Raman spectrometer coupled to an Olympus up-right microscope (Jobin Yvon, France) equipped with a 785 nm diode laser.

A 10x objective with a numerical aperture of 0.25 was used to focus the light onto the surface of the sample. The instrument is equipped with a motorized encoded stage that allows for mapping of samples. Labspec 6.0 software was used for spectral collection, spectral preprocessing and data set processing. Line mapping mode was used to collect spectral maps with steps of 1 μm, and an exposure time of 2 s for each spectrum along distance of 1 μm. Based on the previous research, Spectra were collected in the “fingerprint” region of 600–1800 cm^−1^ using the extended scan mode of the instrument software Labspec6.0 (Horiba Jobin Yvon, France).

### Principle Component Analysis (PCA)

As the reference spectrum, the spectrum of pure bone and ligament were collected where tissue far away from the interface (Fig. 2A,C). The classical least squares (CLS) Fitting algorithm approximates the spectrum at each point in the line by summing together scaled copies of each reference spectrum. The scaling factors are chosen to get the best possible fit to the original map spectrum, and then used to create a map showing the distribution (correlation values) of each reference spectrum. That method can easily distinguished the different components based on the previous provided spectrum.

Based on the data from the Principle Component Analysis, the slope can be measured. Two groups of 18 independent results were collected by the same condition in line map. The spectra were evaluated by the Principle Component Analysis shows how the two different components change. Furthermore, to get more accurate slope based on those data (R^2^ > 0.9), those lines were truncated the flat part (Fig. 3A,B). The result can present how the component change and to compare the two source of tissue.

### Statistical analysis

A test of independent-sample was employed for detecting differences between the 2 groups. For the comparisons, a level of P < 0.05 was considered to be statistically significant. The analysis was performed in SPSS 15 for Windows (SPSS Inc., Chicago, Illinois).

## Additional Information

**How to cite this article**: Wu, Y. *et al*. Evaluation of the Bone-ligament and tendon insertions based on Raman spectrum and its PCA and CLS analysis. *Sci. Rep.* **7**, 38706; doi: 10.1038/srep38706 (2017).

**Publisher's note:** Springer Nature remains neutral with regard to jurisdictional claims in published maps and institutional affiliations.

## Figures and Tables

**Table 1:** Raman shifts/cm^−1^ and assignments of the bands of observed in the interface.

Raman shifts/cm^−1^	Assignment
760	Tryptophan (proteins)
816	Collagen–other proteins, υ C-C protein backbone
855	Proline, hydroxyproline, tyrosine C-C stretching, proline (collagen assignment)
937	Amino acid side chain vibrations of proline and hydroxyproline, as well as a (C-C) vibration of the collagen backbone
960	Symmetric stretching vibration of υ_1_ PO4^3−^ (phosphate of HA)
1002	Phenylalanine (collagen assignment)
1031	Carbohydrate residues of collagen
1100	C-C vibration mode of the gauche-bonded chain
1208	A,T (ring breathing modes of the DNA/RNA bases)-amide III (protein)
1246	Amide III (of collagen)
1314	CH_3_CH_2_ twisting (collagen assignment)
1319	CH_3_CH_2_ twisting (collagen assignment)
1342	CH_3_, CH_2_ wagging (collagen assignment)
1424	Deoxyribose, (B,Z-marker)
1451	CH_2_CH_3_ deformation (collagen assignment)
1663	Proteins, including collagen I
1665	Amide I (of collagen)
