# Corrigendum: Evaluation of the Bone-ligament and tendon insertions based on Raman spectrum and its PCA and CLS analysis

**DOI:** 10.1038/srep43974

**Published:** 2017-03-14

**Authors:** Yang Wu, Yu Dong, Jia Jiang, Haiqing Li, Tongming Zhu, Shiyi Chen

Scientific Reports 7: Article number: 3870610.1038/srep38706; published online: 01 31 2017; updated: 03 14 2017

The Acknowledgements section in this Article was omitted. The Acknowledgements should read: “This work was supported by the National 863 Hi-tech Project (2015AA033703), National Natural Science Foundation of China (NO. 81601882, NO. 81572108) and Specialized Research Fund for the Doctoral Program of Higher Education (NO. 20120071110067)”.

